# Laparoscopic Retrograde Hysterectomy: A Review of Techniques, Indications, and Outcomes

**DOI:** 10.7759/cureus.105429

**Published:** 2026-03-18

**Authors:** Khalid M Akkour

**Affiliations:** 1 Obstetrics and Gynaecology, King Saud University, Faculty of Medicine, Riyadh, SAU

**Keywords:** frozen pelvis, laparoscopic retrograde hysterectomy, learning curve, minimally invasive surgery, operative outcomes

## Abstract

Laparoscopic retrograde hysterectomy (LRH) represents an innovative minimally invasive approach for managing complex pelvic pathology where conventional techniques are contraindicated. This review synthesizes current evidence regarding its techniques, indications, and outcomes. LRH employs a strategic caudal-to-cephalad dissection, prioritizing early identification of ureters and uterine vessels before addressing fundal attachments. Primary indications include severe endometriosis with obliterated cul-de-sac, large uterine size (>300 g), multiple previous surgeries with dense adhesions, and distorted pelvic anatomy. Outcomes demonstrate significant advantages, including reduced conversion rates to laparotomy (0-2.9%), acceptable operative times (66-285 minutes), minimal blood loss (79-450 mL), and short hospital stays (two to six days). The technique maintains low complication rates (2.2-4.7%) despite challenging conditions. However, LRH requires advanced laparoscopic skills and has a substantial learning curve (~65 cases). Widespread adoption necessitates structured training programs and further comparative studies. LRH effectively expands minimally invasive options for complex gynecological surgery, demonstrating particular utility in frozen pelvis scenarios while preserving the benefits of laparoscopy.

## Introduction and background

Hysterectomy is among the most commonly performed major gynecological surgeries globally. In recent decades, there has been significant progress in surgical techniques, with an increasing preference for minimally invasive methods such as total laparoscopic hysterectomy (TLH), which provide benefits like reduced patient morbidity and faster recovery times [1]. However, the laparoscopic method may not be ideal for every patient, especially those with complex pelvic issues, including severe endometriosis, extensive adhesions, or an obliterated cul-de-sac, which complicate the procedure [2].

In complex cases, the presence of adhesive disease and fibrosis significantly alters normal anatomical landmarks and surgical planes. This alteration increases the risk of damaging essential structures such as the ureters, bladder, and bowel during surgical procedures [2]. Moreover, a large and distorted uterus can complicate the movement of instruments and obstruct visibility, making traditional antegrade dissection either risky or unfeasible. These challenges have historically resulted in a higher conversion rate to open laparotomy, diminishing the benefits of minimally invasive techniques for high-risk patients [3].

To overcome the limitations of conventional techniques, the retrograde hysterectomy method was developed. This novel approach alters the standard surgical order by initiating dissection at the cervicovaginal junction or lower uterine segment instead of the uterine fundus. This allows for the early recognition and management of vital structures and uterine vessels before addressing the more challenging regions with dense adhesions near the fundus, potentially enhancing safety and ease in frozen pelvis cases [4-6].

Managing complex pelvic conditions, especially severe endometriosis with an obliterated cul-de-sac, poses considerable surgical difficulties due to altered anatomy and adhesive diseases. Traditional laparoscopic hysterectomy methods often face challenges in these scenarios, heightening the chances of converting to laparotomy and causing visceral injury [6]. The retrograde approach has emerged as a significant advancement, focusing on early management of key structures like the ureters and uterine vessels to improve safety in these high-risk situations [7]. This technique has broadened the possibilities for minimally invasive surgery, serving as a feasible option for patients who might need open surgery [8-12].

## Review

Surgical techniques

Laparoscopic retrograde hysterectomy (LRH) (Table 1) represents a significant change from traditional antegrade techniques, emphasizing early control of anatomical structures and upward dissection to improve safety during intricate pelvic surgeries. The key concept involves reversing the typical surgical order by starting dissection at the cervicovaginal junction instead of the uterine fundus. This method enables prompt recognition and isolation of the ureter and uterine artery, thereby reducing the risk of accidental injury in difficult surgical fields [5]. By postponing the transection of upper uterine attachments, this technique preserves uterine position and counter-traction, enhancing the development of avascular planes around the cervix, particularly in challenging cases [6].

**Table 1:** Key studies on laparoscopic hysterectomy in complex pelvic surgery

Authors	Study Design	Sample Size (n)	Key Findings	Limitations
Nakamura et al. [2]	Technical Report	45	• Reported no ureteral injuries in their series. • Avoided uterine manipulator in cases with severe adhesive anatomy to prevent disturbance of planes.	Focused on technical approach and safety rather than broad outcomes.
Yamamoto et al. [6]	Retrospective Cohort	92	• Feasible for large uteri (≥600g) & obliterated cul-de-sac. • Zero conversions to laparotomy. • Low complication rate (3.3%). • Operative time & blood loss significantly correlated with uterine weight. • Proficiency learning curve: ~65 cases.	Single-center, retrospective design. Selection bias.
Litta et al. [7]	Prospective Study	101	• Fast post-operative recovery: return to activities at ~6 days. • No ureteral injuries reported. • Zero conversions to laparotomy. • Confirmed the safety and efficacy of the reverse technique.	Short-term follow-up data.
Volpi et al. [8]	Case Series	169	• Demonstrated LRH as a standardizable technique in a community hospital setting. • Acceptable complication rate (4.7%), including bladder (2.3%) and ureteral (1.2%) injuries. • Low conversion rate to laparotomy (2.9%).	Heterogeneous patient sample [included benign and malignant cases].
Sinha et al. [9]	Prospective Comparative Study	350 (two groups)	• Compared early vs. conventional uterine artery ligation. • Early ligation (Group A) resulted in shorter operative time (60 vs. 70 min) and lower blood loss (50 vs. 60 mL). • Reduced need for transfusion in the early ligation group. • Conclusion: Establishes the principle of early vascular control, a cornerstone of the LRH technique.	Focused on a single step within TLH, not the full LRH procedure.
Paul et al. [11]	Retrospective Case Series	25	• Early description of LRH for "frozen pelvis" from various causes. • High success rate (96%) with one conversion (4%) due to bladder/bowel injury. • Mean uterine weight: 390.2 g. • Featured retrograde rectal dissection and routine safety tests.	Small sample size. Single-center. High BMI cohort.
Melnyk et al. [13]	Retrospective Cohort	333 (96 with Stage IV endo)	• Comparative study: Patients with obliterated cul-de-sac (n=55) required 93% modified radical hysterectomy vs. 29% without. • Longer median surgical time: 159 min vs. 108 min. • Higher rate of ancillary procedures [ureterolysis, bowel surgery]. • Conclusion: Affirms the complexity of the obliterated cul-de-sac, necessitating specialist care.	Retrospective. Provides context for LRH but not technique-specific outcomes.

The procedure is performed under general anesthesia with the patient in a low lithotomy position. A uterine manipulator may aid uterine mobility and define vaginal fornices, although some techniques avoid its use to prevent disturbing adhered anatomy [1]. Access is achieved via a diamond-shaped multi-port configuration, with a 12-mm umbilical port for the camera and three 5-mm operating ports in the lower abdomen. Pneumoperitoneum is maintained at 10-12 mmHg [6,8].

The retrograde approach begins by incising the peritoneum of the vesicouterine pouch and the anterior leaf of the broad ligament [6,8]. The surgeon then develops the retroperitoneal spaces, paravesical and pararectal, to visualize the ureter as it crosses the iliac vessels into the pelvis. A critical step is systematic ureterolysis and proximal uterine artery ligation through meticulous retroperitoneal dissection. Early ureteral identification is essential to prevent injury [6]. Simultaneously, the uterine artery is skeletonized at its origin from the internal iliac artery and coagulated/transected for early vascular control, minimizing intraoperative bleeding. These maneuvers are particularly valuable in cases with distorted anatomy, such as severe endometriosis or multiple prior surgeries, where traditional landmarks are obscured [2,9].

A defining feature of the retrograde technique is the intentional delay in transecting the upper uterine attachments. The round ligament, infundibulopelvic [or utero-ovarian] ligament, and posterior broad ligament are often left intact initially [2,8]. This preserves uterine position and provides critical counter-traction. The bladder is then dissected off the cervix and upper vagina until the distinct white tissue of the anterior vaginal fornix is visualized [6].

Colpotomy marks the pivotal retrograde step. The uterine manipulator is often replaced with a vaginal delineator (e.g., vagi-pipe or Breisky valve) to elevate the vaginal fornices [2,6]. The colpotomy is initiated anteriorly, using an energy device to make a small transverse incision through the vaginal wall, which is extended circumferentially to the lateral and posterior fornices [6,8]. In cases of obliterated cul-de-sac, traction on the cervix “tents” adhered tissue, making planes more discernible and reducing rectal injury risk [10]. The adhesion is carefully dissected off the posterior cervix and vaginal wall using cold scissors or precise energy dissection, staying close to the uterine serosa to avoid rectal injury [2].

After circumferential colpotomy, the uterus is progressively mobilized retrograde from caudal to cephalad. The remaining parametrial and paracervical tissues, including the uterosacral and cardinal ligaments, are serially coagulated and divided. Since the ureter and uterine artery have been identified and controlled, this phase significantly reduces the risk of hemorrhage or ureteral damage. Finally, the upper attachments, the round and infundibulopelvic ligaments, are divided, culminating in the complete liberation of the uterus [6,8].

Specimen removal is accomplished transvaginally. For larger uteri that cannot be extracted intact, intracorporeal morcellation or posterior vaginotomy may be performed [2]. The vaginal cuff is sutured laparoscopically with absorbable sutures, and meticulous hemostasis is confirmed. The pelvis is irrigated, and some protocols recommend an air-leak test or rectoscopy to rule out unrecognized bowel injury before concluding the procedure. A pelvic drain may be placed at the surgeon's discretion [6].

Indications 

Laparoscopic retrograde hysterectomy is indicated for complex pelvic conditions where conventional hysterectomy poses high risks, particularly severe endometriosis with an obliterated cul-de-sac and dense adhesions. This technique is beneficial for patients with large uterine sizes (≥300 g), multiple prior abdominal surgeries, and cervical or isthmic issues distorting anatomy. It is also applicable in selected benign gynecological cases such as large fibroids, adenomyosis, and chronic pelvic pain, where standard dissection risks injury to the ureters, bladder, or bowel. The retrograde approach is advantageous in frozen pelvis scenarios, allowing surgeons to navigate adherent tissue with direct visualization while minimizing injury to adjacent organs [7-11]. Preoperative imaging, such as MRI, is crucial for identifying obliterated planes and guiding surgical planning, especially when endometriosis involves the rectovaginal septum or parametria [12].

Patient selection criteria 

Ideal candidates for laparoscopic retrograde hysterectomy include patients with complex benign gynecological conditions where conventional approaches pose significant risks. This is particularly relevant for nulliparous women with severe endometriosis, those with multiple previous pelvic surgeries, and patients with large uterine masses hindering safe dissection. This technique is advantageous when anatomical landmarks are obscured but can be accessed via retroperitoneal dissection. Comprehensive preoperative assessment, including detailed pelvic imaging, is crucial for optimal outcomes. Factors such as body mass index, surgical history, and severity of endometriosis must be evaluated. A multidisciplinary evaluation may be needed for cases with suspected bowel or ureteral involvement to enhance safety and outcomes [13,14].

Perioperative outcomes

Laparoscopic retrograde hysterectomy (LRH) consistently demonstrates favorable perioperative outcomes across diverse patient groups and surgical settings, making it a feasible choice for intricate gynecological situations. This procedure upholds the benefits of minimally invasive surgery, even in complicated anatomical conditions that may necessitate converting to laparotomy using traditional methods. The duration of surgeries ranges from 66±15 minutes for uncomplicated benign cases to 285 minutes for more complex situations involving severe endometriosis and significant uterine size. This variation is associated with surgical complexity, especially concerning uterine weight and the severity of adhesive disease. Blood loss remains consistently minimal, with average values from 79.5±138.4 mL to 265 mL, and transfusion rates between 0% and 1.5%. Early identification of the ureters and effective control of the uterine arteries improve hemostasis and lower operative risks. Moreover, the technique results in short hospital stays (median of two to six days) and a swift return to normal activities, illustrating the patient-centered benefits of this approach, even in cases of high complexity [6,9].

Recovery parameters

Postoperative recovery outcomes for laparoscopic radical hysterectomy (LRH) are impressively positive. Patients typically spend a median of two days in the hospital in community settings, while those with more complicated cases may stay for about six days. This is significantly shorter than the recovery associated with traditional laparotomy methods. Most patients are able to return to their daily activities within six to seven days and to their jobs within 12-13 days after surgery. The minimally invasive approach preserves these advantages, even in more challenging cases, with conversion rates to laparotomy remaining notably low at (0-2.9%) in various studies. This quick recovery is particularly beneficial for individuals with severe endometriosis, who generally face extended periods of disability with standard treatments. Factors such as decreased opioid use, faster mobilization, and a lower likelihood of postoperative complications all contribute to the improved recovery experience linked with this surgical technique [13,14].

Learning curve and proficiency

The technical requirements of LRH entail a considerable learning curve, with competence generally reached after around 65 procedures. This learning phase mainly impacts operational time and blood loss during the early adaptation stage. The organized methodology for retroperitoneal dissection and the systematic recognition of anatomical landmarks create a reliable framework that aids in skill development. Although the learning curve can pose challenges for implementation, especially in low-volume centers, the technique shows reliable safety results once mastered [9,11].

Complications and limitations 

Laparoscopic retrograde hysterectomy is generally a safe procedure, but it does come with specific complications that surgeons should be mindful of, particularly during the initial learning period. Complication rates are low, ranging from 2.2% to 4.7%, even in more complicated cases, with bladder injury being the most prevalent major complication (0-2.3%). The retrograde method appears to lower the risk of ureteral injury (0-1.2%) compared with traditional techniques, as it allows for earlier identification and safeguarding of the ureters during dissection. Nonetheless, the technical difficulty of the procedure demands advanced laparoscopic expertise in retroperitoneal dissection and adhesiolysis, which may hinder its widespread use. Careful case selection is essential, as cases involving very large uteri (greater than 1000 g) or frozen pelvises with significant bowel involvement may still require alternative methods, despite the benefits of this technique [8,12].

Discussion

Laparoscopic retrograde hysterectomy (LRH) represents a notable improvement in the surgical treatment of complicated pelvic conditions, broadening the options available to gynecological surgeons for managing difficult anatomical situations previously deemed unsuitable for minimally invasive methods [6,11]. This approach radically alters the surgical methodology by reversing the standard antegrade dissection order. By starting the procedure at the cervicovaginal junction and moving from lower to upper direction, surgeons can first identify and secure essential anatomical references, particularly the ureters and uterine vessels, before addressing the more perilous areas of adhesion near the fundus [7,9]. This systematic approach, from familiar to unfamiliar anatomy, is particularly advantageous in cases of frozen pelvis, where traditional techniques require dissecting through dense, vascular adhesions with limited visibility, thereby significantly increasing the risk of accidental injury to surrounding tissues or blood vessels [13].

The improved safety profile of this method is strongly reinforced by consistently positive outcomes found in the literature. The principle of early vascular control, highlighted by Sinha et al. [9], is fundamental to LRH and directly contributes to the lower median blood loss observed in studies, typically ranging from 50 to 265 mL, even in more complex situations [6,8]. Additionally, the methodical retroperitoneal dissection and ureterolysis allow for the prompt identification of the ureters, which is evident in the notably low rates of ureteral injury (0-1.2%) reported across various studies, even among patients with severe endometriosis and complete cul-de-sac obliteration [2,8]. Perhaps the most compelling evidence of its effectiveness is the significantly low conversion rate to laparotomy (0-2.9%) in these high-risk groups, demonstrating that LRH effectively maintains the advantages of minimally invasive surgery in cases where traditional laparoscopy might not succeed [6-7].

Despite the notable benefits of retrograde hysterectomy, its widespread implementation encounters significant obstacles, mainly due to its technical complexities and the current body of evidence. The procedure requires a high level of expertise in advanced laparoscopic techniques, such as retroperitoneal dissection and complex intracorporeal suturing, which are typically not included in standard gynecological training [14]. As a result, there is a considerable learning curve; Yamamoto et al. [6] found that roughly 65 procedures mark the point at which proficiency in minimizing operative time and blood loss is achieved. This steep learning curve highlights the urgent necessity for organized educational programs, including simulation-based training and mentorship initiatives, to promote safe practice beyond high-volume tertiary centers [11].

In conclusion, while the current evidence from case series and cohort studies is encouraging, it underscores a significant gap in the literature: the lack of high-quality comparative studies. Most of the existing evidence, although consistent across studies such as those by Yamamoto et al. [6] and others, comes mainly from specialized centers, which may introduce selection bias and variations in expertise. There is an urgent need for randomized controlled trials that directly compare LRH with traditional laparoscopic or open methods in complex patient populations. Future research should aim to produce Level I evidence to provide objective validation of its benefits concerning operative outcomes, complications, and long-term recovery. Additionally, initiatives should focus on standardizing technical protocols, improving objective patient selection criteria based on preoperative imaging, and creating validated tools to assess and reduce the learning curve, thus facilitating appropriate implementation across various healthcare settings.

Clinical implications

This review presents laparoscopic retrograde hysterectomy (LRH) as a groundbreaking surgical approach, significantly broadening the minimally invasive options available for patients with intricate pelvic conditions who would have previously required laparotomy. By focusing on the early identification and management of vital structures, LRH clearly improves intraoperative safety, leading to reduced blood loss and lower rates of ureteral injuries, even in cases of frozen pelvis. In clinical practice, this translates to decreased conversions to open surgery, shorter hospital stays, and quicker patient recovery. However, to successfully implement this technique, structured training is essential to address the considerable learning curve, highlighting the importance of referrals to specialized centers and investment in simulation-based proctorship programs to safely expand this advanced skill.

Limitations of the review

This review is constrained by the type of evidence available, which mainly includes retrospective case series and cohort studies from specialized, high-volume centers. The lack of randomized controlled trials (RCTs) directly comparing LRH with traditional methods introduces risks of selection bias and expertise bias, which weaken the reliability of our conclusions. Additionally, the data primarily reflects the outcomes of experienced surgeons, making it difficult to generalize to broader clinical settings. The intricacies and considerable learning curve associated with LRH are not fully reflected in the overall results, which may lead to an underestimation of the procedure's complexity and risks when performed by less experienced practitioners.

Recommendations and future directions

To maximize the potential of laparoscopic retrograde hysterectomy (LRH), structured training and simulation modules are essential to navigate the learning curve safely. Future research should prioritize multicenter randomized controlled trials comparing LRH with conventional techniques in patients with severe endometriosis and frozen pelvis to provide high-level evidence. Additionally, standardizing protocols and establishing imaging-based patient selection criteria are crucial, along with long-term studies to assess outcomes like pelvic floor function and recurrence rates to ensure effectiveness and sustainability.

## Conclusions

Laparoscopic retrograde hysterectomy offers a beneficial minimally invasive method for tackling complex pelvic issues that usually necessitate laparotomy. Its systematic technique for identifying anatomical structures effectively addresses difficulties such as significant adhesions and altered anatomy. To maximize its advantages, there is a need for targeted surgical education, well-defined training programs, and substantial comparative research. With these advancements, retrograde hysterectomy has the potential to become a key procedure in advanced pelvic surgery, broadening treatment options for patients with challenging gynecological problems.

## References

[1] Kantarcı S, Karabulut A, Dağlı U (2025). Minimally invasive hysterectomy approaches: comparative learning curves and perioperative outcomes of robotic versus V-NOTES techniques. J Clin Med.

[2] Nakamura K, Nakayama K, Minamoto T (2019). A novel retrograde approach for total laparoscopic hysterectomy in patients with severe adhesion in the cul-de-sac. Clin Exp Obstet Gynecol.

[3] Kitundu JH, Mashauri HL (2025). Clinical implications of postsurgical adhesions and fibrosis: the role of vitamin C in prevention and control. Health Sci Rep.

[4] Ajabor LN (1976). Evaluating retrograde hysterectomy in the surgery of pelvic gynaecologic diseases in Nigeria. Niger Med J.

[5] Hiramatsu Y (2019). Retrograde abdominal hysterectomy. Surg J (N Y).

[6] Yamamoto M, Yoshida H (2021). Feasibility and safety of total laparoscopic retrograde hysterectomy in a large uterus with obliterated cul-de-sac due to severe endometriosis. Eur J Obstet Gynecol Reprod Biol.

[7] Litta P, Saccardi C, Conte L, Florio P (2013). Reverse hysterectomy: another technique for performing a laparoscopic hysterectomy. J Minim Invasive Gynecol.

[8] Volpi E, Bernardini L, Angeloni M, Cosma S, Mannella P (2014). Retroperitoneal and retrograde total laparoscopic hysterectomy as a standard treatment in a community hospital. Eur J Obstet Gynecol Reprod Biol.

[9] Sinha R, Sundaram M, Nikam YA, Hegde A, Mahajan C (2008). Total laparoscopic hysterectomy with earlier uterine artery ligation. J Minim Invasive Gynecol.

[10] Yoriki K, Kusuki I, Kawamata M, Tarumi Y, Mori T, Kitawaki J (2021). Successful detection of rectal injury during laparoscopic surgery using a rectal probe in a patient with deep endometriosis. J Obstet Gynaecol Res.

[11] Paul PG, Khan S, Sheetal AB, Harneet K, Prathap T, Tony T (2013). Laparoscopic hysterectomy in frozen pelvis—an alternative technique of retrograde adhesiolysis. Gynecol Surg.

[12] Keckstein J, Becker CM, Canis M (2020). Recommendations for the surgical treatment of endometriosis. Part 2: deep endometriosis. Hum Reprod Open.

[13] Melnyk A, Rindos NB, El Khoudary SR, Lee TT (2020). Comparison of laparoscopic hysterectomy in patients with endometriosis with and without an obliterated cul-de-sac. J Minim Invasive Gynecol.

[14] Janssen PF, Brölmann HA, Huirne JA (2011). Recommendations to prevent urinary tract injuries during laparoscopic hysterectomy: a systematic Delphi procedure among experts. J Minim Invasive Gynecol.

